# Quercetin Inhibits Peripheral and Spinal Cord Nociceptive Mechanisms to Reduce Intense Acute Swimming-Induced Muscle Pain in Mice

**DOI:** 10.1371/journal.pone.0162267

**Published:** 2016-09-01

**Authors:** Sergio M. Borghi, Felipe A. Pinho-Ribeiro, Victor Fattori, Allan J. C. Bussmann, Josiane A. Vignoli, Doumit Camilios-Neto, Rubia Casagrande, Waldiceu A. Verri

**Affiliations:** 1 Departamento de Ciências Patológicas, Centro de Ciências Biológicas, Universidade Estadual de Londrina, Rodovia Celso Garcia Cid, Km 380, PR445, Cx. Postal 10.011, 86057-970, Londrina, Paraná, Brasil; 2 Laboratório de Anatomia Patológica, Centro de Ciências de Saúde, Universidade Estadual de Londrina, Avenida Robert Koch, 60, Hospital Universitário, 86039-440, Londrina, Paraná, Brasil; 3 Departamento de Bioquímica e Biotecnologia, Centro de Ciências Exatas, Universidade Estadual de Londrina, Rodovia Celso Garcia Cid, Km 380, PR445, Cx. Postal 10.011, 86057-970, Londrina, Paraná, Brasil; 4 Departamento de Ciências Farmacêuticas, Centro de Ciências de Saúde, Universidade Estadual de Londrina, Avenida Robert Koch, 60, Hospital Universitário, 86039-440, Londrina, Paraná, Brasil; University of Texas Medical Branch at Galveston, UNITED STATES

## Abstract

The present study aimed to evaluate the effects of the flavonoid quercetin (3,3´,4´,5,7-pentahydroxyflavone) in a mice model of intense acute swimming-induced muscle pain, which resembles delayed onset muscle soreness. Quercetin intraperitoneal (i.p.) treatment dose-dependently reduced muscle mechanical hyperalgesia. Quercetin inhibited myeloperoxidase (MPO) and N-acetyl-*β*-D- glucosaminidase (NAG) activities, cytokine production, oxidative stress, cyclooxygenase-2 (COX-2) and gp91^phox^ mRNA expression and muscle injury (creatinine kinase [CK] blood levels and myoblast determination protein [MyoD] mRNA expression) as well as inhibited NFκB activation and induced Nrf2 and HO-1 mRNA expression in the soleus muscle. Beyond inhibiting those peripheral effects, quercetin also inhibited spinal cord cytokine production, oxidative stress and glial cells activation (glial fibrillary acidic protein [GFAP] and ionized calcium-binding adapter molecule 1 [Iba-1] mRNA expression). Concluding, the present data demonstrate that quercetin is a potential molecule for the treatment of muscle pain conditions related to unaccustomed exercise.

## Introduction

The growing recognition of the benefits of physical activities in human health highlights exercises as an essential conduit for rehabilitation programs in a wide variety of chronic inflammatory diseases such as hypertension, atherosclerosis, heart diseases, diabetes, rheumatoid arthritis and cancer [1]. However, sedentary patients with chronic diseases starting exercise protocols are often more susceptible to experience muscle pain, which may lead to discourage the continuity of exercises sessions resulting in abandonment of exercise practicing and lack of exercise health benefits [1].

Untrained individuals experience delayed onset muscle soreness (DOMS). DOMS results from unaccustomed strenuous exercise in untrained or sedentary people. Symptoms of DOMS do not initiate immediately after the exercise session, but rather around 12 h post-exercise, peaking 24–48 h after exercise session [2,3]. DOMS is classified as a type I muscle strain injury, characterized by tenderness or stiffness to palpation and/or movement [4]. The resolution of DOMS symptoms could disappear naturally, or in cases of untrained individuals and patients with chronic debilitating diseases generates severe debilitating pain, leading to restriction of movements [4]. Thus, therapeutic approaches to reduce exercise-induced muscle pain are encouraged and may provide a better quality of life for these individuals. In mice, intense acute swimming induces DOMS-like response by mechanisms involving soleus muscle and spinal cord oxidative stress and cytokine production [1,2].

Flavonoids constitute important components of the human diet. Flavonoid intake ranges between 50 and 800 mg/day depending on the consumption of vegetables and fruits [5]. Indeed, flavonoids have emerged as interesting therapeutic molecules to treat varied inflammatory and painful conditions, especially due to its wide spectrum of biological activities, but additionally because they have fewer adverse effects than other traditional anti-inflammatory/analgesic drugs [5,6]. The flavonoid quercetin (3,3´,4´,5,7-pentahydroxyflavone) is a well-established molecule with anti-inflammatory, antioxidant and analgesic properties [7–12]. For instance, quercetin reduces inflammatory, neuropathic and cancer pain as well as gouty arthritis-related inflammation [7,8,13]. The analgesic effects of quercetin observed in models of nociception were describe to be dependent on many mechanism, including nitric oxide production, activation of ɣ-aminobutyric acid (GABA) and serotonin receptors, opioid like effects, and inhibition of transient receptor potential cation channel subfamily V member 1 (TRPV1)/N-methyl-D-aspartate (NMDA) receptors, cytokine production and oxidative stress [7,8,13].

The effects of oral long-term quercetin supplementation on performance, systemic immune modulations, muscle metabolism or markers of inflammation and oxidative stress in exercise were evaluated in experimental and clinical settings with contrasting results [14–20]. For instance, quercetin is effective in reducing exercise-induced oxidative stress and inflammation in blood samples of unprofessional athletes [18]. Corroborating, quercetin co-ingested with other flavonoids and food components prevents inflammation in trained cyclists 3 days after exercise [16] and confers myocardium protection against intense exercise injury [20]. On the other hand, quercetin did not reduce blood oxidative stress and inflammation or muscle inflammation and damage in post-exercise during clinical trials possibly due to high dose treatment during a long period [14,15,17,19]. Therefore, whether quercetin has beneficial effects in muscle inflammation and oxidative stress is still controversial. Importantly, the analgesic effect and mechanisms of quercetin in exercise-induced muscle pain remain to be investigated.

Therefore, the present study evaluated the analgesic effect of quercetin and its mechanism in muscle mechanical hyperalgesia using a model of intense acute swimming-induced muscle pain in mice. Possible analgesia-related effects of quercetin were also addressed and consisted of evaluating inflammatory- and oxidative stress-related parameters in the soleus and gastrocnemius muscles as well as spinal cord.

## Methods

### Animals

The experiments were performed on male Swiss mice, weighing between 20-25g from State University of Londrina, PR, Brazil. Mice were housed in standard clear plastic cages with free access to water and food, light/dark cycle of 12/12 h and controlled temperature. Mice were maintained in the vivarium of the Department of Pathological Science of State University of Londrina for at least two days before the experiments. Mice were used only once and were acclimatized to the testing room at least 1 hour before the experiments, which was conducted during the light cycle. At the end of experiments, mice were anesthetized with isoflurane 3% to minimize suffering (Abbott Park, IL, USA) and terminally killed by cervical dislocation followed by decapitation. The animal condition was monitored daily and at indicated time points during the experiments. No unexpected animal deaths occurred during this study. Animals’ care and handling procedures were in accordance with the International Association for Study of Pain (IASP) guidelines and were approved by the Institutional Ethics Committee for Animal Research of State University of Londrina under the process number 13279.2011.76. All efforts were made to minimize the number of animals used and their suffering.

### Test compounds

The compounds used in this study were saline solution (NaCl 0.9%; Frenesius Kabi Brasil Ltda, Aquiraz, CE, Brazil), isoflurane (Abbott Park, IL, USA), dimethyl sulfoxide (DMSO; Sigma-Aldrich, St. Louis, MO, USA) and quercetin (95% purity, Acros, Fair Lawn, NJ, USA).

### General experimental procedures

**Fig 1:**
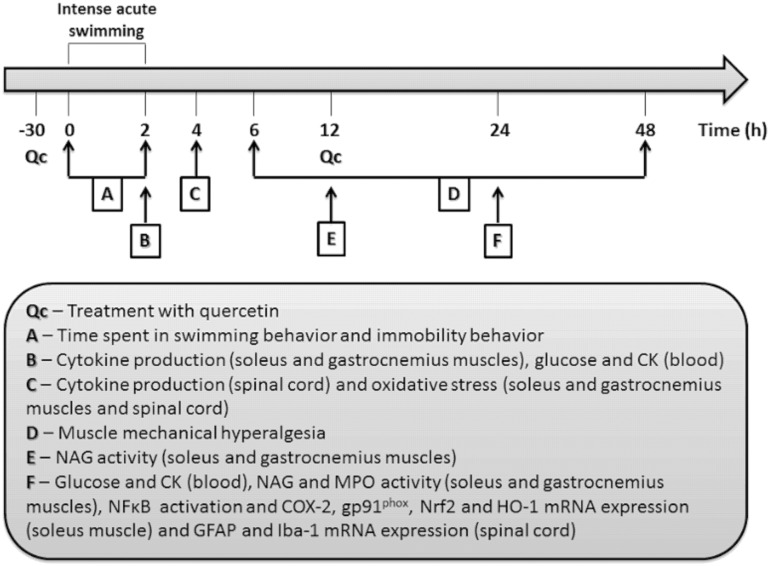
Schematic protocol. Mice were treated with quercetin 30 min before and 12 h after (depending on the experiment), and were submitted to intense acute swimming session. During the swimming session (0–2 h), time spent in swimming behavior and immobility behavior were determined; cytokine production in the soleus and gastrocnemius muscles and glucose and CK levels in blood were assessed immediately after the session (2 h); cytokine production in the spinal cord and oxidative stress in the soleus and gastrocnemius muscles and spinal cord were assessed 2 h after the session (4 h); muscle mechanical hyperalgesia were evaluated 6–48 h after the session; NAG activity was assessed 12 h after the session as part of time-response experiment; glucose and CK levels in blood, MPO and NAG activity (time-response experiment as well as the time period chosen), NFκB activation, COX-2, gp91^phox^, Nrf2 and HO-1 mRNA expression in the soleus and gastrocnemius muscles and GFAP and Iba-1 mRNA expression in the spinal cord were assessed 24 h after the session.

**Fig 2:**
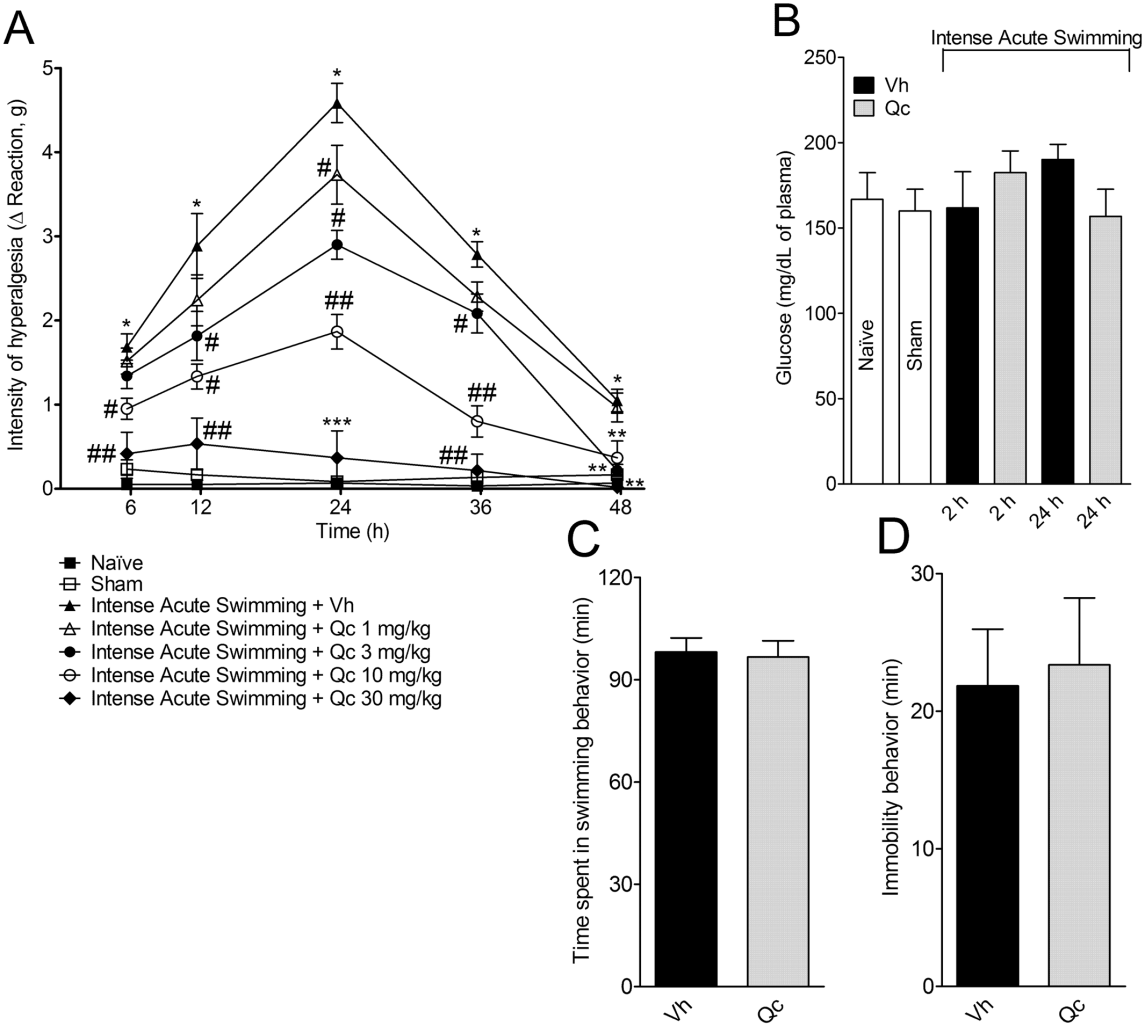
Quercetin reduces in a dose-dependent manner intense acute swimming-induced muscle mechanical hyperalgesia and did not affect glucose levels, time spent in swimming behavior or immobility behavior during the intense acute swimming session. Mice received vehicle (2% DMSO in saline) or quercetin (1–30 mg/kg, i.p.) 30 min before plus reinforcements 12 h after the intense acute swimming session. The intensity of muscle mechanical hyperalgesia was evaluated 6–48 h after the intense acute swimming session (Panel A). Glucose plasmatic levels were determined immediately after and 24 h (peak of the hyperalgesia) after the swimming session (Panel B). Time spent in swimming behavior (Panel C) and immobility behavior (Panel D) were measured during the period of 2 h of the intense acute swimming session in quercetin (30 mg/kg, i.p., 30 min before) and vehicle treated groups. Results are presented as intensity of hyperalgesia (Δ reaction, in grams), glucose (mg/dL of plasma), time spent in swimming behavior and immobility behavior in minutes (Panels A-D) (*n* = 6 mice per group per experiment, representative of two independent experiments). *P<0.05 compared with naïve and sham groups, #P<0.05 compared with vehicle group, **P<0.05 compared with vehicle and 1 mg/kg groups, ##P<0.05 compared with vehicle, 1 and 3 mg/kg groups, ***P<0.05 compared with vehicle, 1, 3 and 10 mg/kg groups (Two-way ANOVA followed by Tukey’s *post hoc*).

Fig 1 shows the time points of treatments and sample collection, which were based on previous studies or selected in experiments shown in the present study [1,2,21]. Mice received vehicle (Vh, 2% DMSO in saline, 200 μg/mL) or quercetin (Qc, 1–30 mg/kg, diluted in 2% DMSO in saline, 200 μg/mL) by intraperitoneal (i.p.) route 30 min before swimming session plus reinforcements 12 h after, depending on the sample collection time point and were submitted to one session of intense acute swimming of 120 min [1,2,21]. The doses of 1, 3 and 10 mg/kg of quercetin were used only in the dose-response experiment presented in Fig 2. Naïve and sham (30 sec exposure to water) animals were used as control groups. After intense acute swimming session, the following tissues, parameters and time points were used for evaluation of: muscle mechanical hyperalgesia (6–48 h after swimming session); time spent in swimming behavior/immobility behavior during swimming session; plasmatic levels of glucose (2 and 24 h after swimming session); myeloperoxidase (MPO) and N-acetyl-*β*-D-glucosaminidase (NAG) activities in the soleus and gastrocnemius muscle (24 and 12 and 24 h after swimming session, respectively); cytokine (TNF-α, IL-1β and IL-10) production in the soleus and gastrocnemius muscle (immediately after swimming session); antioxidant capacity by 2,2´-azinobis (3-ethylbenzothiazoline-6-sulphonic acid) (ABTS) and reduced glutathione (GSH) levels as well as superoxide anion production (nitroblue tetrazolium [NBT] reduction) and lipid peroxidation (thiobarbituric acid reactive substances [TBARS]) levels in the soleus and gastrocnemius muscles (2 h after swimming session); NFκB activation (24 h), and cyclooxygenase-2 (COX-2), gp91^phox^, Nrf2, HO-1, and myoblast determination protein (MyoD) mRNA expression were determined in the soleus muscle (24 h after swimming session); creatine kinase (CK) levels in the blood (2 and 24 h after swimming session); cytokine (TNF-α, IL-1β and IL-10) production, GSH and TBARS levels in spinal cord (L4-L6; 2 h after swimming session); and glial fibrillary acidic protein (GFAP) and ionized calcium-binding adapter molecule 1 (Iba-1) mRNA expression in the spinal cord (L4-L6; 24 h after swimming session). The experimental design and times described above were based on previous studies [1,2,21].

### Intense acute swimming session

Mice were placed in a glass box (45×28×25 cm, divided into six compartments) with approximately 20 liters of water at 31° ± 1°C. Each mouse was placed in one compartment and all swam the same time, except by sham animals, which swam 30 seconds. Mice in the swimming group were exposed to water for one session with duration of 120 min. The muscle mechanical hyperalgesia was evaluated 6–48 h after the swimming session. The swimming session period was defined in previous studies that demonstrated a swimming time-dependent muscle hyperalgesia [1,2,21]. Briefly, in the first study standardizing this model, we determined the swimming time necessary to induce significant muscle hyperalgesia using 30 min of swimming at five consecutive days or swimming only once for 30 min, 1h and 2 h of swimming. The 2 h swimming induced a hyperalgesic response with temporal profile (muscle hyperalgesia 6–48 h after swimming session, peaking at 24 h; for review, see ref. 2) resembling clinical delayed onset muscle soreness (DOMS). Furthermore, the intensity of muscle hyperalgesia induced by this protocol is amenable by inhibition of pro-hyperalgesic cytokines such as TNFα and IL-1β as well as it is enhanced by IL-10 deficiency and inhibited by IL-10 treatment. Therefore, the intensity of muscle hyperalgesia achieved in this protocol allows observing inhibition and enhancement, thus, further supporting it is balanced and not excessive [1,2,21]. This model of exercise also avoids stress- or hypoalgesia- related to water temperature, and focuses on exercise-induced hyperalgesia [1,2,21]. Importantly, mice floated during approximately 19% of the session. Therefore, this swimming behavior is intentional and likely in an attempt to find a scape, thus mice choose how much they swim. The welfare of mice was always a focus and, therefore, an Experimenter monitored the swimming sessions. Swimming sessions were also recorded to determine the swimming/floating ratio of each mouse.

### Evaluation of muscle mechanical hyperalgesia

Muscle mechanical hyperalgesia was tested in mice as previously reported [22]. The test consisted of evoking a hind paw flexion reflex with a hand-held force transducer (electronic von Frey anesthesiometer; Insight, Ribeirão Preto, SP, Brazil) adapted with a 0.5 mm^2^ contact area polypropylene tip. Muscle mechanical hyperalgesia was measured at four time points after the intense acute swimming session (6, 12, 24 and 48 h) always in the right hind limb of mice to determine the intensity of muscle pain after the session. Briefly, mice were placed in individual acrylic cages (12×10×17 cm) with wire grid floors allowing free moving for acclimatization 15–30 min before the start of testing. Mice were awake and no anesthesia was administrated. The total period in which animals remained in acrylic cages did not exceed 45 min in each evaluation time (0, 6, 12, 24 and 48 h) considering the periods of acclimatization and measurements. For the measurements of muscle mechanical hyperalgesia, the edge of tip was brought into contact to the paw tissue and an upward pressure was applied. The pressure applied to hind paw surface induces an articular movement on the ankle joint (dorsiflexion), leading to stretch the Aquilles tendon, which in turn, promotes an exaggerated muscle movement response (movement-induced hyperalgesia) when there is sensitization of nociceptors of calf muscle [1,2,21]. The end point was characterized by the removal of the paw followed by clear flinching movements. Muscle distension is sufficient to trigger muscle nociceptive responses. After the paw withdrawal, the intensity of the pressure was recorded automatically. The value of the response was an average of three measurements. The results are expressed by delta (Δ) withdrawal threshold (in g) calculated by subtracting the mean measurements (indicated time points) after stimulus from the baseline measurements. The basal mechanical withdrawal threshold was 8.8±0.1 g (mean ± SEM between the groups, 6 mice per group per experiment) before intense acute swimming session. There was no difference of basal mechanical withdrawal thresholds between groups in the same experiment.

### Cytokine production

Samples were homogenized in 500 μl (soleus muscle and spinal cord) or 700 μl (gastrocnemius muscle) of the appropriate buffer containing protease inhibitors. TNFα, IL-1β and IL-10 production was determined by enzyme-linked immunosorbent assay (ELISA) using eBioscience kits (Affymetrix, San Diego, CA, USA) [1,2]. Briefly, 96-well plates were coated overnight at 4°C with an immunoaffinity-purified polyclonal sheep antibody specifically for each cytokine tested. After blocking the plates, recombinant murine TNF-α, IL-1β and IL-10 standards at various dilutions and the samples were added in duplicate and incubated overnight at 4°C. Rabbit biotinylated immunoaffinity-purified antibodies anti-TNF-α, anti-IL-1β, anti-IL-10 were added, followed by incubation at room temperature for 1 h. Finally, 50 μL of avidin-HRP (1:5000 dilution) was added to each well; after 30 min, the plates were washed and the color reagent o-phenylenediamine (200 μg/well; Sigma) was added. After 15 min, the reaction was interrupted with 1 M H_2_SO_4_ and measured at 450 nm. The results were expressed as picograms (pg) of cytokine per 100 mg of tissue.

### NFκB activation assay

Samples were homogenized in ice-cold lysis buffer (Cell Signaling Technology, Beverly, MA, USA). The homogenates were centrifuged (200 g × 10 min × 4°C) and the supernatants used to assess the levels of total and phosphorylated NF-κB p65 subunit by ELISA using PathScan kits (Cell Signaling Technology, Beverly, MA, USA) according to the manufacturer’s instructions [23]. The results are presented as the sample ratio of NFκB activation (total p65/phospho-p65) per milligram of soleus muscle measured at 450 nm.

### MPO activity

The leukocyte migration to soleus and gastrocnemius muscles induced by intense acute swimming was evaluated using the MPO kinetic-colorimetric assay as described previously [1]. Briefly, samples of skeletal muscles were collected in 50 mM K_2_HPO_4_ buffer (pH 6.0) containing 0.5% hexadecyl trimethylammonium bromide (HTAB) and kept at −86°C until use. Samples were homogenized using a Polytron (PT3100) and centrifuged at 16,100 *g* in 4°C for 2 min. Then, 10 μl of the resulting supernatant were mixed with 200 μl of 50 mM phosphate buffer pH 6.0, containing 0.167 mg/ml O-dianisidine dihydrochloride and 0.0005% hydrogen peroxide and assayed spectrophotometrically for MPO activity determination at 450 nm (Multiskan GO Microplate Spectrophotometer, Thermo Fischer Scientific, Vantaa, Finland). The MPO activity of samples was compared to a standard curve of neutrophils, and the results were presented as MPO activity (numbers of total neutrophils x 10^10^/mg of soleus and gastrocnemius muscles).

### NAG activity

NAG activity was determined by an adapted colorimetric method previously described [24]. Briefly, 20 *μ*L of the supernatant obtained from MPO activity procedure was added in a 96-well plate followed by the addition of 80 μL of 50 mM phosphate buffer, pH 6.0. The reaction was initiated by the addition of 2.24 mM 4-nitrophenyl N-acetyl-β-D-glucosaminide. The plate was incubated at 37°C for 10min, and the reaction was stopped by the addition of 100 μL of 0.2 M glycine buffer, pH 10.6. NAG enzymatic activity was determined at 400 nm (Multiskan GO Microplate Spectrophotometer, Thermo Fischer Scientific, Vantaa, Finland), and results were presented as NAG activity (macrophages x 10^4^/mg of soleus and gastrocnemius muscles).

### ABTS assay

Samples were homogenized in ice-cold buffer containing 1.15% KCl using a Polytron (PT3100) and centrifuged at 200 g in 4°C for 10 min [24,25]. The total antioxidant capacity of the supernatant was measured by ABTS assay, which is based on the ability of the tissue antioxidant molecules to quench ABTS radical cation (ABTS^⋅+^), a bluegreen chromophore with characteristic absorption at 734 nm. ABTS^⋅+^ was produced by reacting ABTS stock solution (ABTS dissolved in water to a 7mM concentration) with 2.45 mM potassium persulfate (final concentration) and allowing the mixture to stand in the dark at room temperature for 12–16 h before use. For the study, ABTS^⋅+^ solution was diluted in phosphate buffer pH 7.4 to reach an absorbance of 0.8 (±0.02) at 734 nm. Briefly, 10 μL of the samples was added to 1 mL of the diluted ABTS^⋅+^ solution; samples were vortex-mixed and allowed to stand for 6 min, to be measured by spectrophotometer at 734 nm. A standard curve was prepared using different concentrations of Trolox and results were presented as nmol of Trolox equivalent/mg of soleus and gastrocnemius muscles.

### GSH assay

Samples were homogenized in ethylenediaminetetraacetic acid (EDTA) 0.02 M, and homogenates treated with 2 mL H_2_O milli Q plus 0.5 mL of trichloroacetic acid (TCA) 50%. In the next step, homogenates were centrifuged (1,500 *g*, 15 min) and the resultant supernatant was added to 2 mL of a solution containing Tris 0.4 M (pH 8.9) plus 50 mL of dithionitrobenzoic acid (DTNB). Then, after 5 min, the measurements were performed using a spectrophotometer at 412 nm (Multiskan GO Microplate Spectrophotometer, Thermo Fischer Scientific, Vantaa, Finland) [1]. The results were presented as GSH (mmols per miligrams of soleus and gastrocnemius muscles and spinal cord).

### Superoxide anion production

Samples were homogenized with 500 μL of saline, and 50 μL of the homogenate was placed in a 96-well plate, followed by the addition of 100 μl of nitroblue tetrazolium (NBT solution, 1 mg/mL) and incubation for 1 h at 37°C. The supernatant was carefully removed, and the precipitated formazan was then solubilized by adding 120 μL of 2 M KOH and 140 μL of DMSO. Superoxide anion production was determined spectrophotometrically by the reduction of the redox dye NBT [21–25]. Readings were performed at 600 nm (Multiskan GO Microplate Spectrophotometer, Thermo Fischer Scientific, Vantaa, Finland). Protein quantification was used for data normalization, and the results were presented as NBT reduction (OD/mg of protein of soleus and gastrocnemius muscles).

### Lipid peroxidation determination

TBARS levels were used to determine lipid peroxidation in skeletal muscle and spinal cord samples [24]. For this assay, TCA (10%) was added to the homogenate to precipitate proteins followed by centrifugation (1,000 *g*, 3 min, 4°C). The protein-free supernatant was then separated, and thiobarbituric acid (0.67%) was added. The mixture was kept in water bath for 15 min at 100°C. Malondialdehyde (MDA), an intermediate product of lipid peroxidation, was determined by difference between absorbance at 535 and 572 nm (Multiskan GO Microplate Spectrophotometer, Thermo Fischer Scientific, Vantaa, Finland). Protein quantification was used for data normalization, and the results were presented as TBARS (nmol MDA/mg of protein of soleus and gastrocnemius muscles and spinal cord).

### Reverse transcription and quantitative polymerase chain reaction

Reverse transcription and quantitative polymerase chain reaction (qPCR) were performed as previously described [25]. Samples of the soleus muscle and spinal cord were homogenized in trizol reagent. Subsequently, total RNA was isolated according to the manufacturer’s guideline. The purity of total RNA was measured spectrophotometrically and the wavelength absorption ratio (260/280) was between 1.8 and 2.0 for all preparations. Reverse transcription of total RNA to cDNA and qPCR were carried out using GoTaq^®^ 2-Step RT-qPCR System (Promega) and specific primers. qPCR reaction was performed in StepOnePlus^™^ Real-Time PCR System (Applied Biosystems^®^). The relative gene expression was measured using the comparative 2^-(ΔΔCq)^ method. The expression of Glyceraldehyde 3-phosphate dehydrogenase (GAPDH) mRNA was used as reference gene to normalize data. The primers used in the study were: *COX-2*, forward: 5´-GTGGAAAAACCTCGTCCAGA-3´, reverse: 5´-GCTCGGCTTCCAGTATTGAG-3´; *gp91*^*phox*^, forward: 5´-AGCTATGAGGTGGTGATGTTAGTGG-3´, reverse: 5´-CACAATATTTGTACCAGACAGACTTGAG-3´; *Nrf2*, forward: 5´-TCACACGAGATGAGCTTAGGGCAA-3´, reverse: 5´-TACAGTTCTGGGCGGCGACTTTAT-3´; *HO-1*, forward: 5´-CCCAAAACTGGCCTGTAAAA-3´, reverse: 5´-CGTGGTCAGTCAACATGGAT-3´; *MyoD*, forward: 5´-ACTTTCTGGAGCCCTCCTGGCA-3´, reverse: 5´-TTTGTTGCACTACACAGCATG-3´; *GFAP*, forward: 5´-GGCGCTCAATGCTGGCTTCA-3´, reverse: 5´-TCTGCCTCCAGCCTCAGGTT-3´; *Iba-1*, forward: 5´-ATGGAGTTTGATCTGAATGGAAAT-3´, reverse: 5´-TCAGGGCAGCTCGGAGATAGCTTT-3´; *GAPDH*, forward: 5´-CATACCAGGAAATGAGCTTG-3´, reverse: 5´-ATGACATCAAGAAGGTGGTG-3´.

### Plasmatic concentrations of glucose and CK

Blood samples were centrifuged at 3,300 *g* at 4°C for 5 min, and the resultant plasma was assayed for glucose and CK levels (Dimension^®^ Clinical Chemistry System; Siemens, Erlangen, Germany). Quantification of glucose and CK concentrations was done using a spectrophotometer according to the manufacturer’s instructions [1,2,21]. Naive and sham groups were not fed during the period in which vehicle and quercetin treated animals swam, representing the basal values of non-exercised animals.

### Statistical analysis

Results are presented as means ± standard error mean (SEM) of measurements made on six mice in each group per experiment and are representative of two independent experiments. Two-way repeated measure analysis of variance (ANOVA) followed by Tukey’s *post hoc* was used to compare the groups in multiple time points after the intense acute swimming session. Analyzed factors were treatments, time and time versus treatment interaction. On the other hand, one-way ANOVA followed by Tukey’s *post hoc* were performed for data of single time point. Data were analyzed using the statistical software GraphPad Prism 5 (Graphpad Software Inc., La Jolla, CA, USA). Statistical differences were considered significant when P < 0.05.

## Results

### Quercetin treatment inhibits intense acute swimming-induced muscle mechanical hyperalgesia in a dose-dependent manner, and does not affect glucose levels, time spent in swimming behavior or immobility behavior during the intense acute swimming session

Fig 1 summarizes the treatment protocols and time points in which each analysis was performed. Fig 2A shows that intense acute swimming induced significant muscle mechanical hyperalgesia that increased in a time-dependent manner from 6–24 h until reaching its peak at 24^th^ h. After this period, muscle mechanical hyperalgesia decreased gradually until the 48^th^ h. Treatment with quercetin reduced muscle mechanical hyperalgesia in a dose-dependent manner. The doses of 1 and 3 mg/kg of quercetin inhibited muscle mechanical hyperalgesia at 24 h and 12–36 h, respectively. The dose of 10 mg/kg of quercetin inhibited muscle mechanical hyperalgesia between 6–48 h with significant differences compared with two lower doses of quercetin (1 and 3 mg/kg) between 24–36 h, and at 48 h compared with the lower dose of quercetin (1 mg/kg). The dose of 30 mg/kg of quercetin abolished muscle mechanical hyperalgesia between 6–48 h with significant differences compared with the three lower doses of quercetin tested (1–10 mg/kg) at the peak of hyperalgesia (24 h). Therefore, the dose of quercetin of 30 mg/kg was selected for the next sets of experiments. Further confirming that the intense acute swimming protocol is not stressful for animals [1,2,21], plasmatic concentrations of glucose were unaltered immediately after the session (2 h) and at the peak of muscle mechanical hyperalgesia (24 h) (Fig 2B). Additionally, the time spent in swimming (Fig 2C) and immobility (Fig 2D) behaviors during the swimming session were equivalent between vehicle and quercetin treated animals. These results evidence that the analgesic effect of quercetin was not dependent on diminishing the swimming behavior. The analgesic mechanisms of quercetin were evaluated in the next sets of experiments focusing on inflammation- and oxidative stress-related events.

### Quercetin inhibits intense acute swimming-induced MPO and NAG activity in the soleus muscle, but not in the gastrocnemius muscle

**Fig 3:**
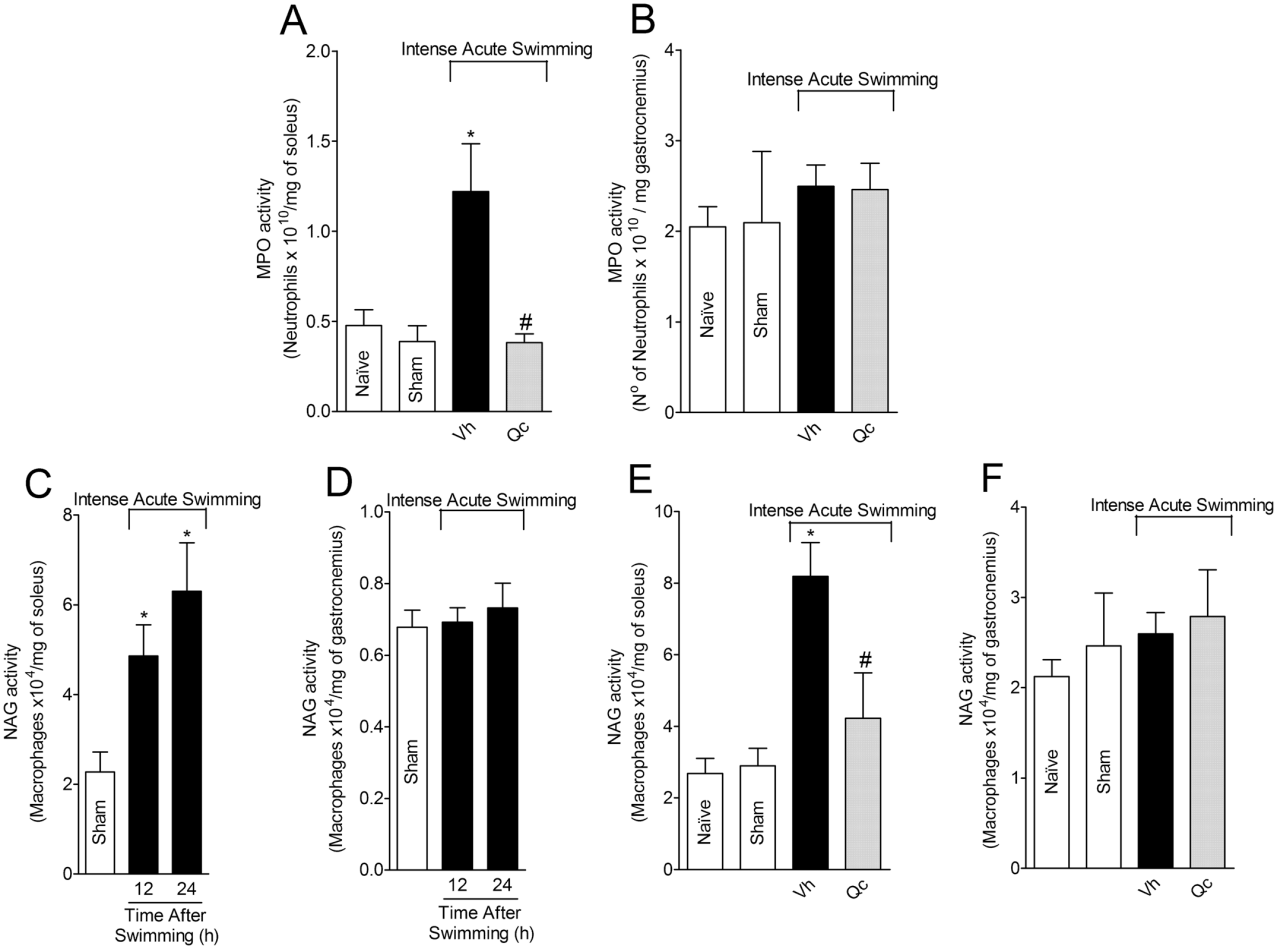
Quercetin reduces intense acute swimming-induced increase in MPO and NAG activities in the soleus muscle but not in the gastrocnemius muscle. Mice were treated with vehicle or quercetin (30 mg/kg, i.p.) 30 min before plus reinforcements 12 h after the intense acute swimming session. MPO (Panels A and B) and NAG (Panels C and D) activities were measured 24 h after the intense acute swimming session. Results are presented as MPO (Neutrophils x 10^10^) and NAG (Macrophages x 10^4^) activity per milligram of the soleus and gastrocnemius muscles (*n* = 6 mice per group per experiment, representative of two independent experiments). *P<0.05 compared to the naïve and sham groups, #P<0.05 compared with vehicle group (One-way ANOVA followed by Tukey’s *post hoc*).

The MPO and NAG activity were determined as indirect markers of neutrophils/macrophages and macrophages counts, respectively [24]. The intense acute swimming-induced increase of MPO activity in the soleus muscle was abolished in quercetin treated group (Fig 3A). Intense acute swimming did not induce significant increase of MPO activity in the gastrocnemius muscle [1,2,21], thus, it could not be altered by quercetin treatment (Fig 3B). In the soleus muscle, NAG activity increased significantly at 12 h and peaked 24 h after the swimming session compared to sham group (Fig 3C). Intense acute swimming did not induce NAG activity increase in the gastrocnemius muscle compared to sham group (Fig 3D). Therefore, the 24 h time point was selected to determine the effect of quercetin on intense acute swimming-induced NAG activity. Quercetin treatment reduced intense acute swimming-induced NAG activity in the soleus muscle (Fig 3E). No alteration was detected in the gastrocnemius muscle (Fig 3F).

### Quercetin inhibits intense acute swimming-induced cytokine production (TNF-α, IL-1β and IL-10) in the soleus muscle, but not in the gastrocnemius muscle

**Fig 4:**
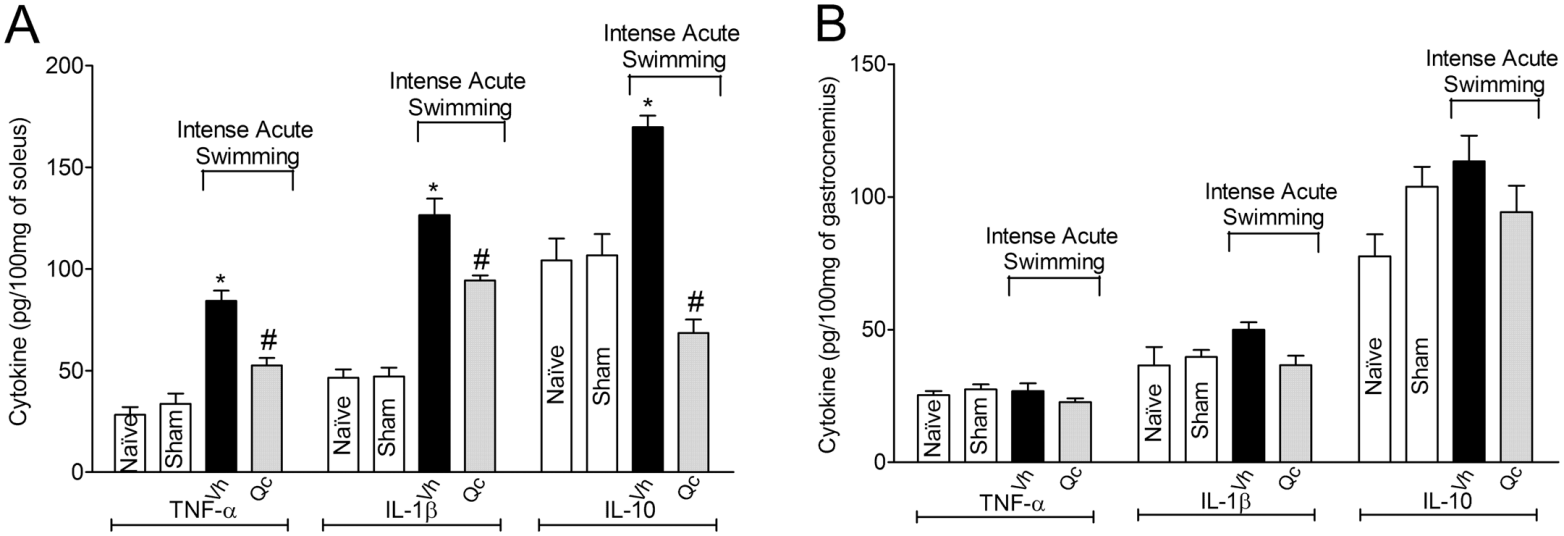
Quercetin reduces intense acute swimming-induced TNF-α, IL-1β and IL-10 production in the soleus muscle, but not in the gastrocnemius muscle. Mice were treated with vehicle or quercetin (30 mg/kg, i.p.) 30 min before intense acute swimming session. The TNF-α, IL-1β and IL-10 concentration in the soleus (Panel A) and gastrocnemius (Panel B) muscles were quantified immediately after the end of intense acute swimming session by ELISA. Results are presented as picograms (pg) per 100 mg of soleus and gastrocnemius muscles samples (*n* = 6 mice per group per experiment, representative of two independent experiments). *P<0.05 compared to the naïve and sham groups, #P<0.05 compared with vehicle group (One-way ANOVA followed by Tukey’s *post hoc*).

Intense acute swimming induced significant TNF-α, IL-1β and IL-10 production in the soleus muscle similar to previous data [1,2,21], and quercetin inhibited the cytokine production (Fig 4A). No alteration was detected in the gastrocnemius muscle (Fig 4B).

### Quercetin inhibits intense acute swimming-induced oxidative stress in the soleus muscle, but not in the gastrocnemius muscle

**Fig 5:**
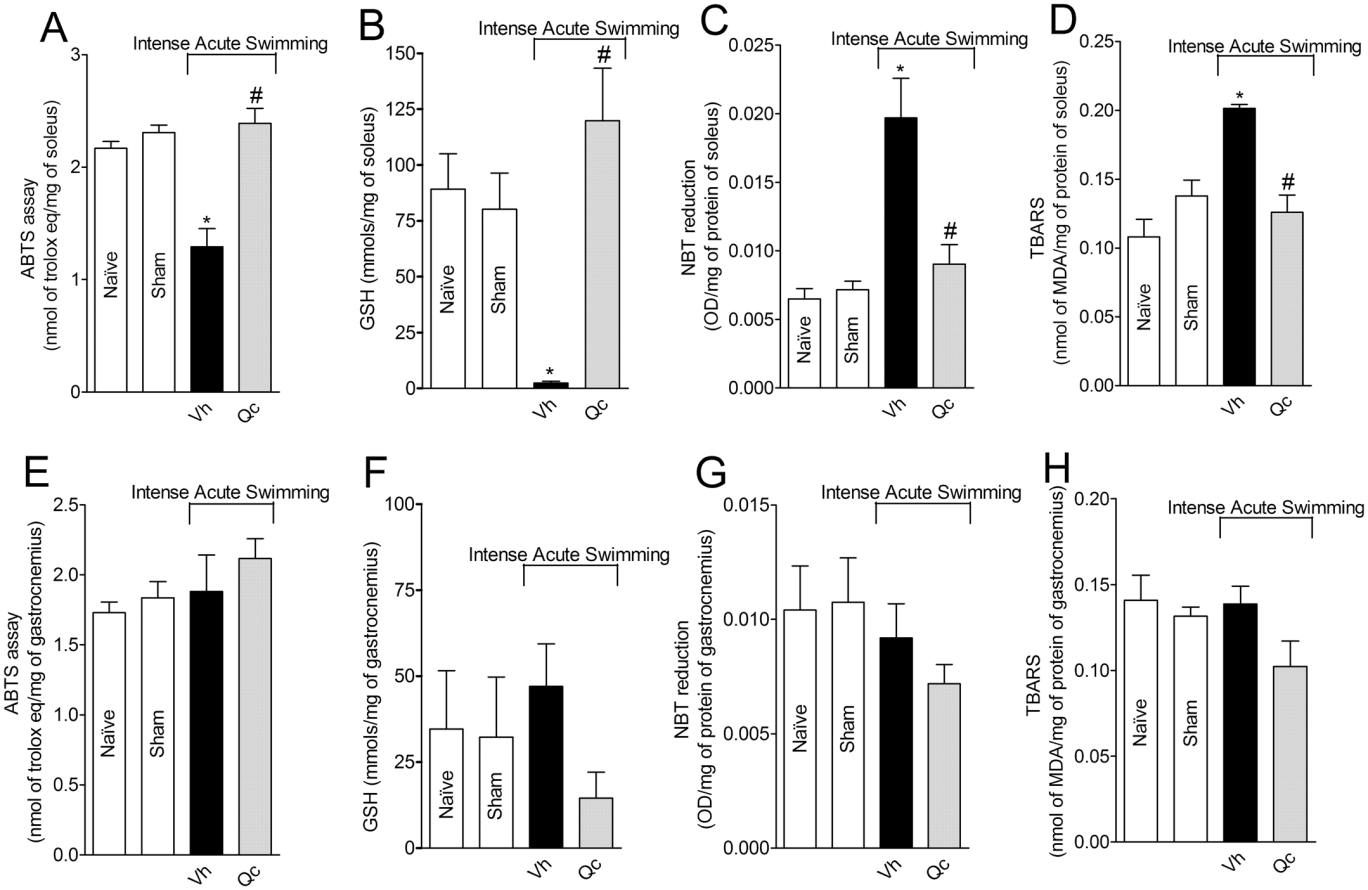
Quercetin reduces intense acute swimming-induced oxidative stress in the soleus muscle, but not in the gastrocnemius muscle. Mice were treated with vehicle or quercetin (30 mg/kg, i.p.) 30 min before intense acute swimming session. The ABTS, GSH, NBT and TBARS assays in the soleus (Panels A-D) and gastrocnemius (Panels E- H) muscles were quantified in samples collected 2 h after the end of intense acute swimming session. Results are presented as ABTS assay (nmol of trolox eq/mg), GSH (mmols/mg), NBT reduction (OD/mg of protein) and TBARS (nmol of MDA/mg of protein) of soleus and gastrocnemius muscles (*n* = 6 mice per group per experiment, representative of two independent experiments). *P<0.05 compared to the naïve and sham groups, #P<0.05 compared with vehicle group (One-way ANOVA followed by Tukey’s *post hoc*).

Intense acute swimming reduced the antioxidant capacity, as observed by ABTS assay and GSH levels (Fig 5A and 5B, respectively), and increased superoxide anion production and lipid peroxidation, as observed by NBT reduction and TBARS levels (Fig 5C and 5D, respectively) in the soleus muscle. Treatment with quercetin significantly re-established the antioxidant capacity (ABTS and GSH) as well as reduced the oxidative stress (superoxide anion production and lipid peroxidation) (Fig 5A–5D). No alteration was detected in the gastrocnemius muscle regarding antioxidant capacity (ABTS and GSH levels) and oxidative stress (superoxide anion production and lipid peroxidation) in the gastrocnemius muscle (Fig 5E–5H).

### Quercetin inhibits intense acute swimming-induced COX-2 and gp91^phox^ mRNA expression in the soleus muscle

**Fig 6:**
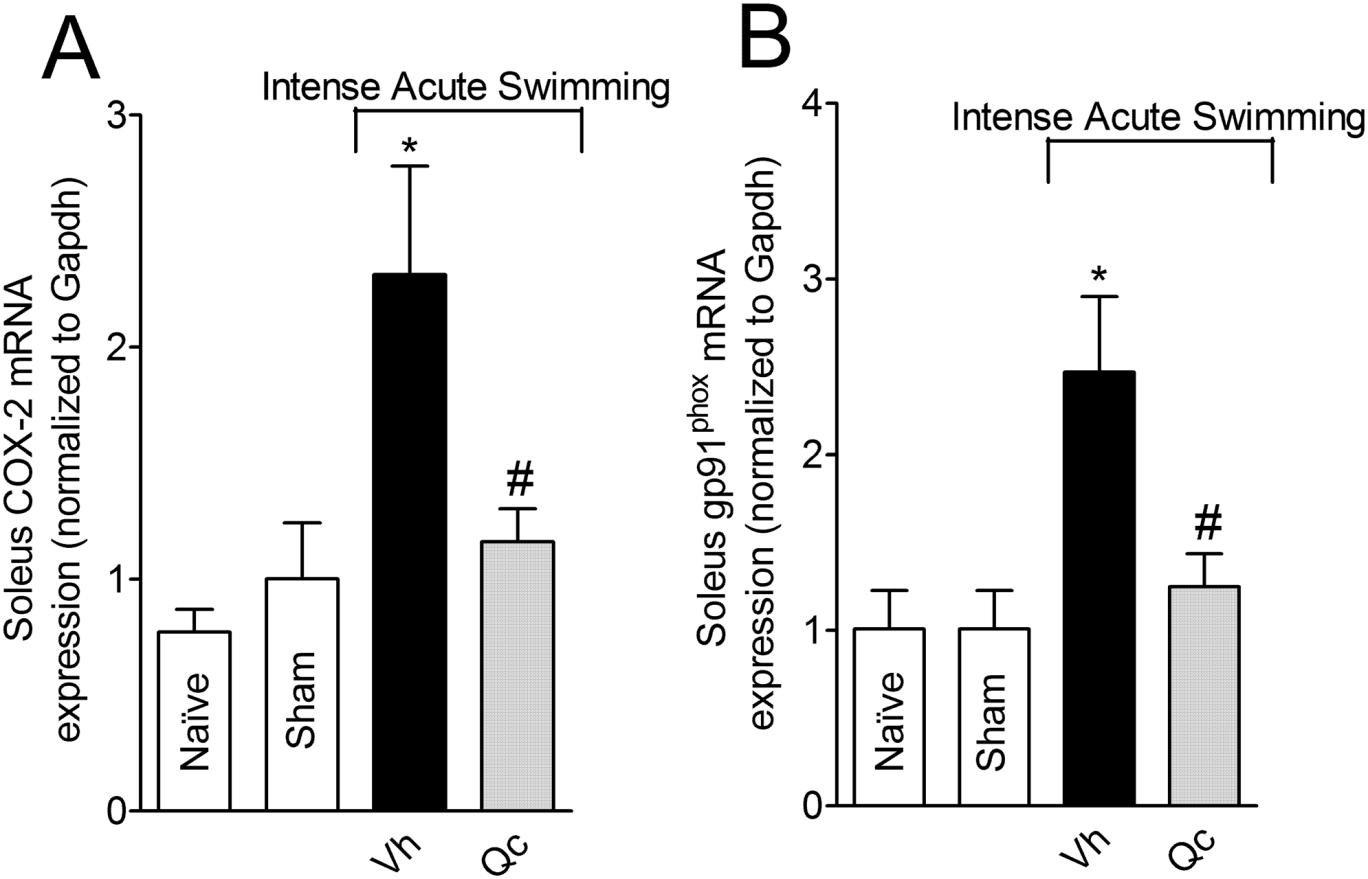
Quercetin reduces intense acute swimming-induced COX-2 and gp91^phox^ mRNA expression in the soleus muscle. Mice were treated with vehicle or quercetin (30 mg/kg, i.p.) 30 min before plus reinforcements 12 h after the intense acute swimming session. Samples of soleus muscle were collected 24 h after the intense acute swimming session. COX-2 (Panel A) and gp91^phox^ (Panel B) mRNA expression were determined by qPCR. Results are presented as COX-2 and gp91^phox^ mRNA expression (normalized to Gapdh) (*n* = 6 mice per group per experiment, representative of two independent experiments). *P<0.05 compared to the naïve and sham groups, #P<0.05 compared with vehicle group (One-way ANOVA followed by Tukey’s *post hoc*).

Intense acute swimming induced significant increase of COX-2 and gp91^phox^ mRNA expression (Fig 6A and 6B, respectively) compared to negative control groups. On the other hand, quercetin reduced intense acute swimming-induced COX-2 and gp91^phox^ mRNA expression (Fig 6A and 6B, respectively).

### Quercetin inhibits NFκB activation and induces Nrf2 and HO-1 mRNA expression after swimming session in the soleus muscle

**Fig 7:**
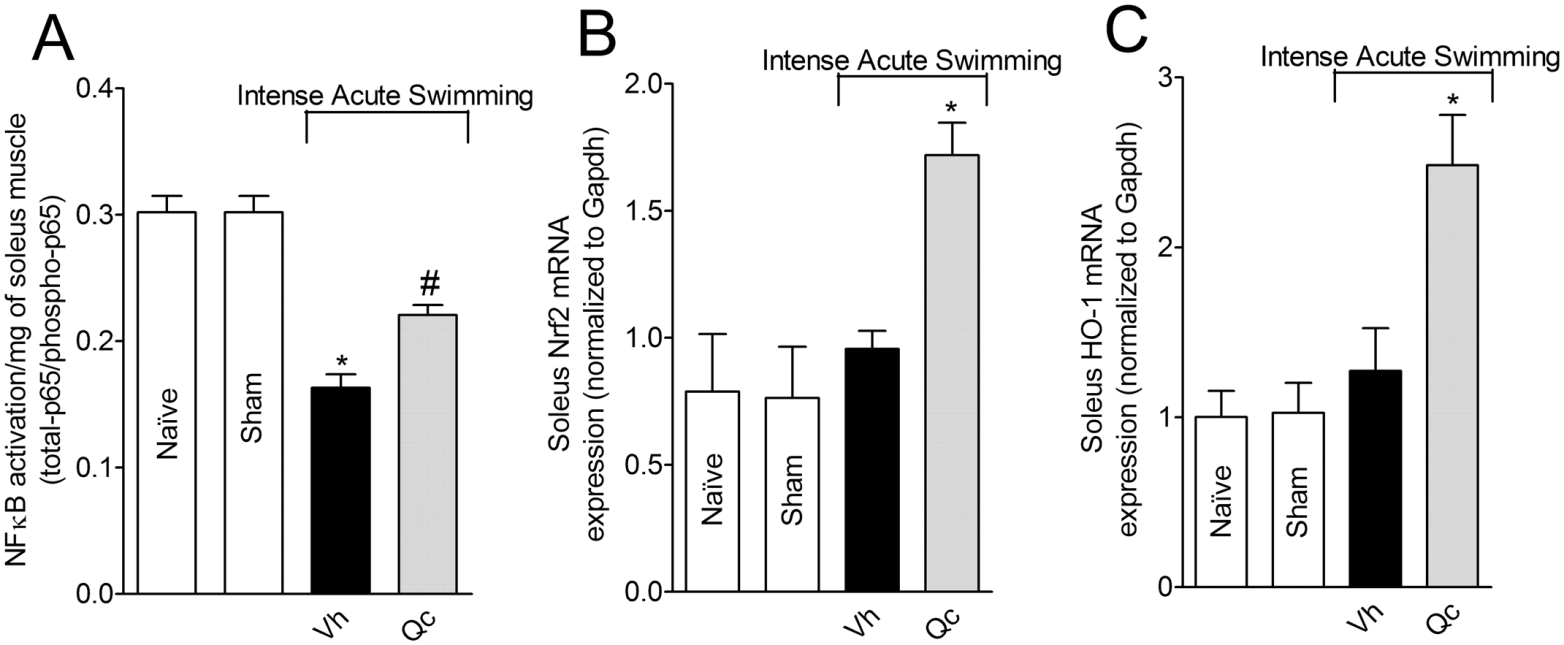
Quercetin inhibits NFκB activation and induces Nrf2 and HO-1 mRNA expression in the soleus muscle after intense acute swimming. Mice were treated with vehicle or quercetin (30 mg/kg, i.p.) 30 min before plus reinforcements 12 h after the intense acute swimming session. NFκB activation (total NFκB/phosphorylated NFκB ratio, Panel A), and Nrf2 (Panel B) and HO-1 (Panel C) mRNA expression in the soleus muscle were assessed 24 h after the intense acute swimming session. Results are presented as NFκB activation (total-p65/phosphotrilated-p65 ratio)/mg of soleus muscle, and Nrf2 and HO-1 mRNA expression (normalized to Gapdh) (*n* = 6 mice per group per experiment, representative of two independent experiments). *P<0.05 compared to the vehicle treated group (One-way ANOVA followed by Tukey’s *post hoc*).

Quercetin inhibited intense acute swimming-induced NFκB activation (total NFκB/phosphorylated NFκB ratio, Fig 7A). Intense acute swimming did not alter Nrf2 and HO-1 mRNA expression compared to control groups. On the other hand, quercetin treatment induced significant increase of Nrf2 and HO-1 mRNA expression (Fig 7B and 7C, respectively).

### Quercetin reduces intense acute swimming-induced myocyte injury

**Fig 8:**
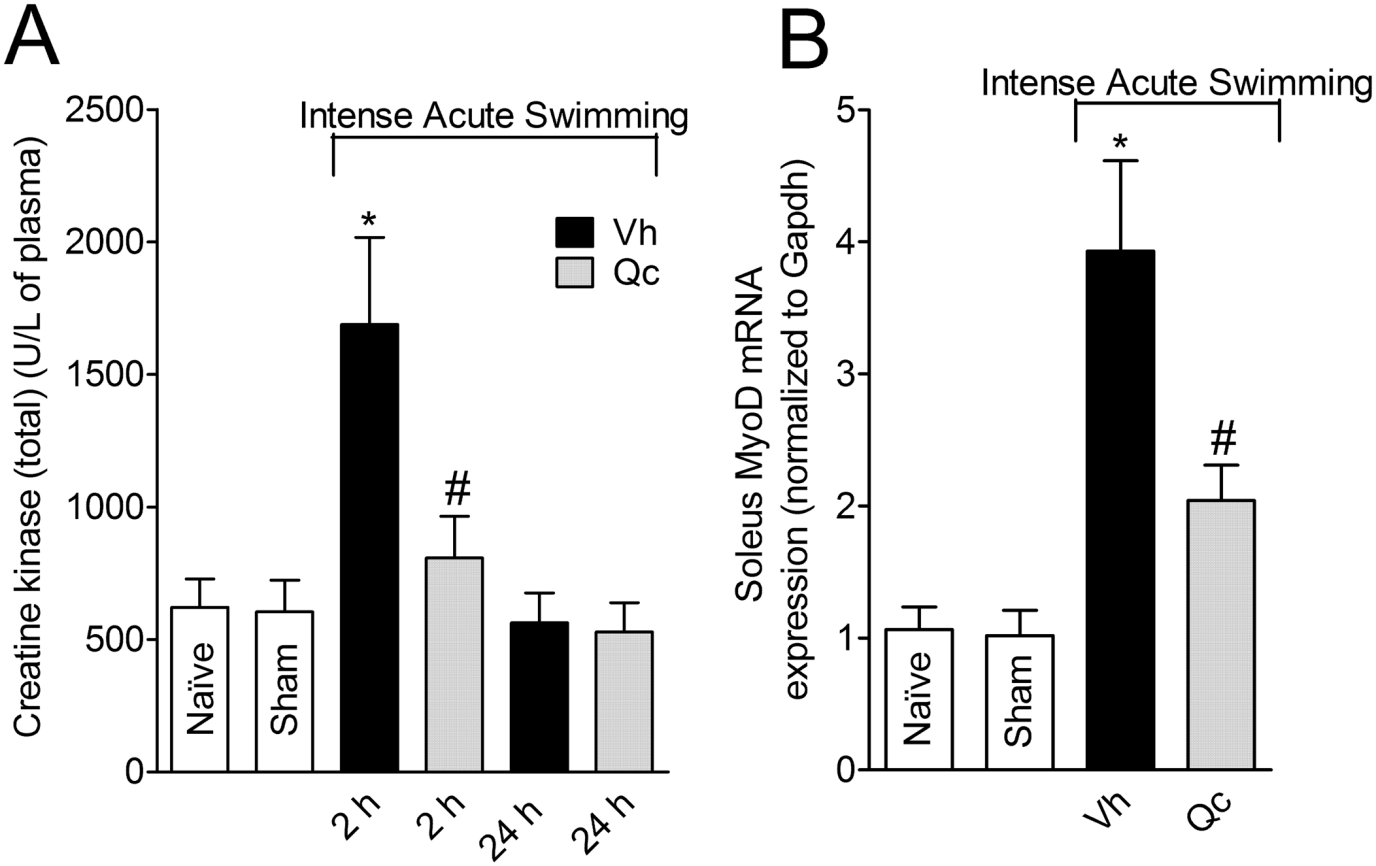
Quercetin reduces intense acute swimming-induced increases in plasmatic concentrations of CK and MyoD mRNA expression in the soleus muscle. Mice were treated with vehicle or quercetin (30 mg/kg, i.p.) 30 min before plus reinforcements 12 h after the intense acute swimming session. At 2 or 24 h after the intense acute swimming session, blood samples were collected for determination of plasmatic levels of CK (Panel A). Samples of the soleus muscle were collected 24 h after the intense acute swimming session for evaluation of MyoD mRNA expression (Panel B). Results are presented as creatine kinase (total) (U/L of plasma) and MyoD mRNA expression (normalized to Gapdh) (*n* = 6 mice per group per experiment, representative of two independent experiments). *P<0.05 compared to the naïve and sham groups, #P<0.05 compared with vehicle group (One-way ANOVA followed by Tukey’s *post hoc*).

Intense acute swimming induced a significant increase in plasmatic levels of CK immediately after the session (2 h), returning to the basal levels at 24^th^ h (Fig 8A) corroborating earlier data [1,21]. Quercetin inhibited intense acute swimming-induced CK blood concentration increase at 2 h (Fig 8A). Intense acute swimming also induced MyoD mRNA expression, which was inhibited by quercetin (Fig 8B).

### Quercetin inhibits intense acute swimming-induced spinal cord cytokine production (TNF-α, IL-1β and IL-10), oxidative stress, and glial cells (astrocyte and microglia) activation

**Fig 9:**
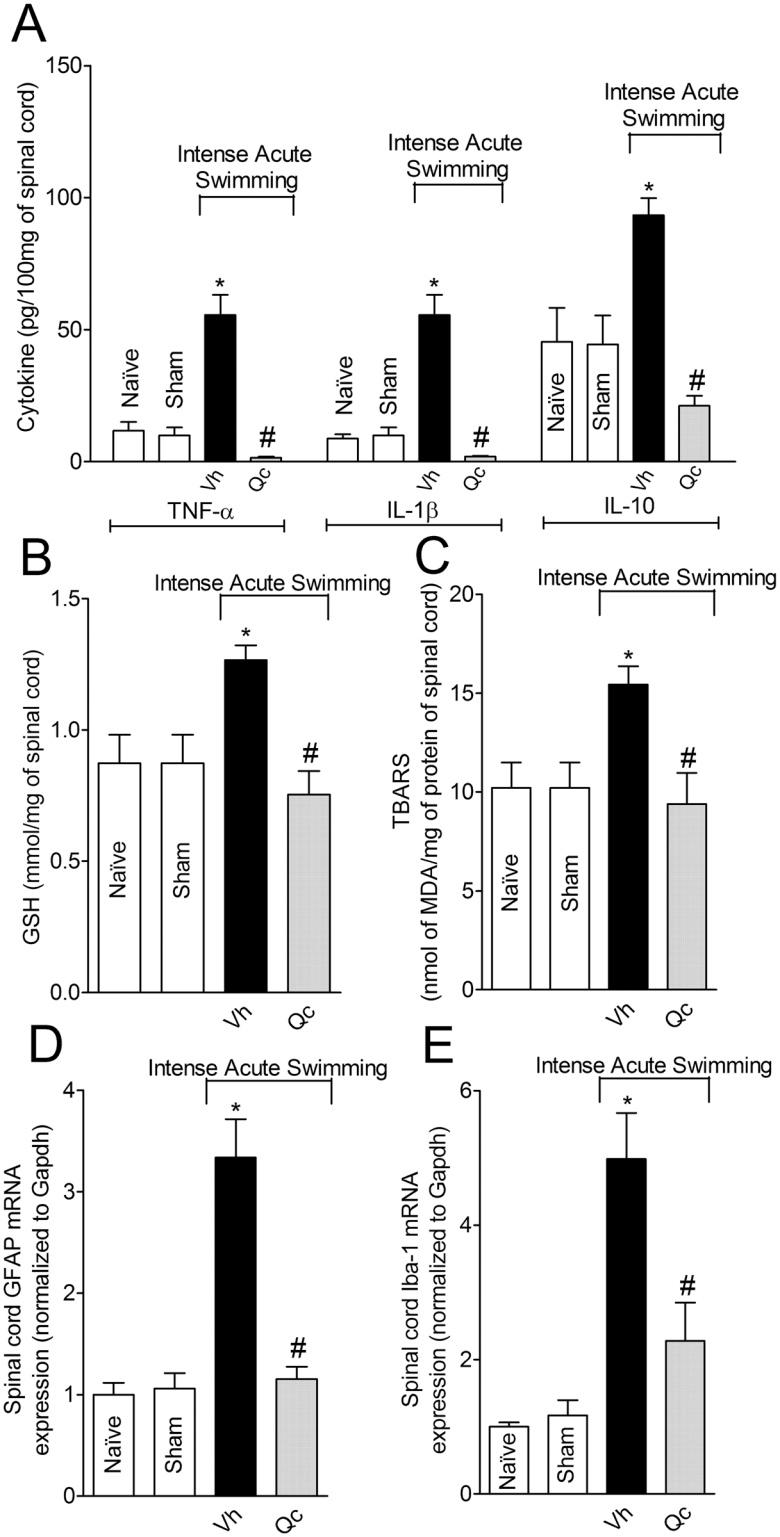
Quercetin reduces intense acute swimming-induced increases in cytokine production, oxidative stress and glial cells activation in the spinal cord. Mice were treated with vehicle or quercetin (30 mg/kg, i.p.) 30 min before (for cytokine production and oxidative stress determination) plus reinforcements 12 h after the intense acute swimming session (for glial cells activation assessment). Samples of the spinal cord (L4-L6) were collected 2 h after the intense acute swimming session for evaluation of cytokine production (TNF-α, IL-1β and IL-10) (Panel A) and oxidative stress (GSH and TBARS) (Panels B and C). GFAP and Iba-1 mRNA expression in spinal cord samples was determined 24 h after the swimming session (Panels D and E). Results are presented as cytokines (picograms per 100 mg), GSH (mmol/mg) and TBARS (nmol of MDA/mg of protein) of spinal cord samples, and GFAP and Iba-1 mRNA expression (normalized to Gapdh) (*n* = 6 mice per group per experiment, representative of two independent experiments). *P<0.05 compared to the naïve and sham groups, #P<0.05 compared with vehicle group (One-way ANOVA followed by Tukey’s *post hoc*).

Intense acute swimming induced TNF-α, IL-1β and IL-10 production in the spinal cord, which was significantly reduced by quercetin treatment (Fig 9A). In muscle samples, four parameters of antioxidant defenses (ABTS and GSH) and oxidative stress (superoxide anion and TBARS) were evaluated. However, spinal cord samples need to be pooled for analysis (spinal cord pool of three mice), therefore, we selected GSH and TBARS parameters to reduce the number of mice. GSH levels (Fig 9B) and lipid peroxidation (Fig 9C) increased in the spinal cord after intense acute swimming session. Quercetin treatment significantly inhibited intense acute swimming-induced increase of GSH levels and reduced lipid peroxidation (Fig 9B and 9C, respectively). In agreement with the production of inflammatory mediators and oxidative stress in the spinal cord, intense acute swimming induced a significant increase of GFAP and Iba-1 mRNA expression in the spinal cord [26], which was reduced by quercetin treatment (Fig 9D and 9E, respectively).

## Discussion

The present results demonstrate that quercetin treatment inhibited in a dose-dependent manner the intense acute swimming-induced muscle hyperalgesia. The antinociceptive effect of quercetin was accompanied by inhibition of intense acute swimming-induced NFκB activation and induction of Nrf2/HO-1 signaling pathway in the soleus muscle. In agreement with the modulation of NFκB and Nrf2, quercetin inhibited intense acute swimming-induced leukocyte recruitment (MPO and NAG activities), cytokine production (TNF-α, IL-1β and IL-10), oxidative stress, COX-2 and gp91^phox^ mRNA expression and tissue damage in the soleus muscle (CK blood concentration and MyoD mRNA expression). Moreover, quercetin also inhibited intense acute swimming-induced spinal cord neuro-inflammation as observed by reduced cytokine production, oxidative alterations and glial cells activation.

As evidenced previously, soleus muscle has a major role in intense acute swimming-induced mechanical hyperalgesia compared to gastrocnemius muscle [1,2,21]. The physiological characteristics of different types of skeletal muscle fibers might be determinant for the prominent role of the soleus muscle in the present model. Soleus muscle is composed mostly by slow type fibers (type I and type IIa), characterized by the resistance to fatigue, and abundant capillary supply, myoglobin concentration and mitochondria. Soleus muscle is also a highly oxidative muscle. Slow type I fibers are predominantly recruited during endurance exercise compared to the other muscle fiber types predominant in the gastrocnemius muscle (IIa and IIb) [2,27]. It is possible that intense acute swimming not only activates the soleus muscle, but also the gastrocnemius muscle. However, it is likely that during the swimming session, the gastrocnemius muscle plays a role in the beginning of the exercise session due to the low fatigue resistance properties of the fast fibers, which rapidly enter into fatigue in the early stages of swimming. As the soleus muscle is intensely active during swimming session period, it produces an extensive amount of chemical mediators which contribute more markedly to the events observed after intense acute swimming session [1,2]. Nonetheless, the participation of the gastrocnemius muscle in intense acute swimming-induced muscle mechanical hyperalgesia cannot be completely ruled out since variables such as the intensity of exercise and time point of evaluation may influence the results [2].

An important point to highlight is that time spent in swimming behavior and immobility behavior during swimming session did not differ among the studied groups. These observations support and validate the behavior, immune and biochemical analysis demonstrated here, indicating that the different responses observed in non-treated and quercetin treated groups cannot be attributed to variations in the swimming time. Furthermore, the similar levels of plasmatic glucose in all groups immediately after and also in the peak of hyperalgesia (24 h) corroborates our previous studies demonstrating no alteration of glucose and cortisol levels, reinforcing the notion that intense acute swimming-induced muscle mechanical hyperalgesia is not related to inducing animal stress [1,2,21].

The present findings are consistent with previous evidence demonstrating that the analgesic effect of quercetin depends on inhibiting the production of pro-hyperalgesic cytokines and oxidative stress in inflammation [8]. It is possible that these anti-inflammatory and antioxidant effects of quercetin are dependent on each other or even independent of each other. Quercetin presents hydroxyl groups capable of donating hydrogen and accepting electrons, which will be stabilized by quercetin benzenic rings. Therefore, the chemical struture of quercetin explains its antioxidant effects. During inflammation there is production of free radicals, which can activate inflammatory signaling pathways leading to cytokine production. Thus, an antioxidant effect can result in an anti-inflammatory effect. On the other hand, the anti-inflammatory effect of quercetin is not entirely or solely related to its antioxidant structures. In fact, there is a clear structure relationship for the antioxidant effects of flavonoids, but not to their inhibition of intracellular signaling pathways related to inflammation, cell survival and proliferation. If the anti-inflammatory and antioxidant effects of quercetin were directly and entirely interdependent, the anti-inflammatory effect would follow the same antioxidant structure relationship [5]. Nevertheless, the interaction of anti-inflammatory and antioxidant mechanisms is possible since cytokines induce free radical production and vice-versa [25]. The anti-inflammatory effects of quercetin have a great contribution of modulating the NFκB pathway. Quercetin inhibits NFκB binding to DNA and NFκB translocation to the nucleus without interefering with IκB degradation [28]. And a protocol of exhaustive swimming induces NFκB activation in the soleus muscle [29]. These data are in accordance with the present study, which demonstrates that quercetin inhibits intense acute swimming-induced NFκB activation. The intense acute swimming-induced muscle hyperalgesia depends on the balance of pro-inflammatory and anti-inflammatory cytokines [1,2,21]. There is evidence that quercetin can induce IL-10 production and this mechanism may explain at least in part its anti-inflammatory effects [30,31]. The present observation that quercetin inhibited intense acute swimming-induced IL-10 production was unexpected. A possible explanation is that as quercetin inhibited oxidative stress and pro-hyperalgesic cytokine production (TNF-α and IL-1β), the endogenous production of IL-10 to limit hyperalgesia was not necessary [21,32].

Quercetin also reduced MPO and NAG activities in the soleus muscle. These results are in line with the reduction of cytokine production (TNF-α and IL-1β) in the soleus muscle and with the chemoattractive role of TNF-α and IL-1β over neutrophils [1,2,33]. For instance, TNF-α activates both neutrophils and macrophages [1,30,34]. Thus, inhibiting TNF-α and IL-1β production reduces the recruitment and activation of these cells. In addition to inhibiting intense acute swimming-induced soleus muscle decrease of GSH and ABTS and soleus muscle increase of superoxide anion and TBARS, quercetin inhibited COX-2 and gp91^phox^ mRNA expression. COX-2 produces hyperalgesic prostanoids and superoxide anion [35]. gp91^phox^ is a subunit of nicotinamide adenine dinucleotide phosphate oxidase (NADPH oxidase) membrane-bound enzyme complex that produces superoxide anion [24]. The products of COX-2 and NADPH oxidase induce hyperalgesia [36,37]. The inhibition of COX-2 mRNA expression observed in the present study corroborates previous data, which demonstrated that quercetin down-regulates COX-2 expression *in vitro* [38]. This is an important data since COX-2 mediates muscle mechanical hyperalgesia, and its product prostagladin E_2_ (PGE_2_) directly sensitizes nociceptors in inflammatory hyperalgesia and muscle pain [36,39,40]. Decrease in gp91^phox^ mRNA expression by quercetin treatment is consistent with the inhibition of oxidative stress considering that NADPH oxidase produces superoxide anion, which lead to oxidative damage through the generation of the deleterious free radical hydroxyl [41]. Thereby, controlling oxidative stress (inhibition of gp91^phox^ mRNA expression) and PGE_2_-induced muscle nociceptors activation (inhibition of COX-2 mRNA expression) might account to the anti-nociceptive and anti-inflammatory effects of quercetin observed in the present study. Importantly, NFκB is a master regulator of the production/expression of cytokines, COX-2 and gp91^phox^ [42–47].

Quercetin also induced Nrf2 mRNA expression and its downstream target HO-1 24 h after intense acute swimming session corroborating previous data showing these effects of quercetin in other experimental systems using varied cell lines [40,48]. This is also an analgesic, antioxidant and anti-inflammatory pathway [23,24,49,50]. Nrf2 also induces GSH production [51], therefore, this result is in agreement with the quercetin prevention of GSH depletion. Thus, the present Nrf2/HO-1 mRNA data support the behavioral and biochemical evidence of the antioxidant and anti-inflammatory effects of quercetin in intense acute swimming-induced muscle mechanical hyperalgesia.

The present study detected that intense acute swimming session leads to increased MyoD mRNA expression 24 h after the session, which is a reliable indicator of satellite cells activation into myoblasts and indicates muscle injury. MyoD is a key transcription factor necessary for the developmental myogenesis and muscle repair events, and could be detected within 6 h following muscle injury [52,53,54]. Quercetin treatment reduced MyoD mRNA expression, indicating that this flavonoid lessened muscle injury. This myoskeletal protective-like effect of quercetin was suported by the reduction of intense acute swimming-induced increase of CK blood concentration.

Peripheral inflammation induces neuronal and glial cells activation in the dorsal root ganglia and spinal cord contributing to neuronal plasticity, and acute and chronic pain [55–57]. Muscle pain conditions also activate astrocytes and microglia in peripheral and central nervous systems [58–61]. For instance, exhaustive swimming exercise induce astrocytes activation in the hippocampus of rats [62]. Considering that these glial cells are well known sources of pro-inflammatory cytokines and reactive oxygen species in central nervous system, the quercetin inhibition of intense acute swimming-induced astrocytic (GFAP mRNA expression) and microglial (Iba-1 mRNA expression) activation in the spinal cord may explain the reduced cytokine production and oxidative stress in this foci [61,63,64]. The reduction of spinal cord glial cells activation, cytokine production and oxidative stress will result in reduced hyperalgesia [27,28,39]. In this sense, it is hypothezized that quercetin inhibits peripheral events induced by intense acute swimming session resulting in the reduced nociceptive transmission of primary afferent neurons innervating the muscle to the spinal cord neurons and glial cells. Nevertheless, there is also evidence that quercetin metabolites reach the cerebrospinal fluid after peripheral treatment [65–67]. Quercetin also induces neuroprotective effects via inhibition of oxidative stress and inflammation in a traumatic brain injury model was also demonstrated [68]. Considering the information described above, local actions of quercetin in the spinal cord could not be disproved.

Of note, intense acute swimming induced an increase of spinal GSH levels opposing to peripheral soleus muscle data in which GSH levels were reduced, which may seem to be a contradiction. However, there is evidence that peripheral stimuli may induce peripheral oxidative stress as well as enhance spinal cord antioxidant defences [1,69,70]. It is likely that the stimuli effect was more proeminent at the primary foci, which had its antioxidant defences overwhelmed. On the other hand, as the spinal cord is a distant foci, it was activated through activation of the primary nociceptive neuron and a minor oxidative stress occurred in the spinal cord and the induction of antioxidant defences could be noticed [1,69,70] as observed by the enhancement of GSH levels. As quercetin inhibited the peripheral inflammation, oxidative stress and muscle damage, the spinal cord activation was reduced, including the induction of GSH production.

## Conclusion

This study demonstrates a novel pharmacological effect of quercetin, the inhibition of DOMS-like-induced pain in mice. Quercetin inhibited intense acute swimming-induced muscle mechanical hyperalgesia, leukocyte recruitment / activation, cytokine production (TNF-α, IL-1β and IL-10), NFκB activation, oxidative stress (improvements in the antioxidant mechanisms / reduction in the superoxide anion generation and lipid peroxidation), COX-2 and NADPH oxidase subunit (gp91^phox^) mRNA expression, and myocyte damage as well as induced Nrf2 and HO-1 mRNA expression. In the spinal cord, quercetin inhibited astrocytes and microglia activation and consequently, cytokine production and oxidative stress, leading to reduced neuro-inflammation. In this sense, the present study suggests that quercetin may be considered a promising molecule to control endurance exercise-induced muscle pain, and to avoid patients who need physical activity to leave exercise programs due to DOMS.

## References

[1] Borghi SM, Zarpelon AC, Pinho-Ribeiro FA, Cardoso RD, Cunha TM, Alves-Filho JC, et al Targeting interleukin-1beta reduces intense acute swimming-induced muscle mechanical hyperalgesia in mice. J Pharm Pharmacol. 2014;66: 1009–1020. 10.1111/jphp.12226 24697255

[2] Borghi SM, Zarpelon AC, Pinho-Ribeiro FA, Cardoso RD, Martins-Pinge MC, Tatakihara RI, et al Role of TNF-alpha/TNFR1 in intense acute swimming-induced delayed onset muscle soreness in mice. Physiol Behav. 2014;128: 277–287. 10.1016/j.physbeh.2014.01.023 24518865

[3] Graven-Nielsen T, Arendt-Nielsen L. Induction and assessment of muscle pain, referred pain, and muscular hyperalgesia. Curr Pain Headache Rep. 2003;7: 443–451. 14604503 10.1007/s11916-003-0060-y

[4] Cheung K, Hume P, Maxwell L. Delayed onset muscle soreness: treatment strategies and performance factors. Sports Med. 2003;33: 145–164. 12617692 10.2165/00007256-200333020-00005

[5] Verri WA Jr, Vicentini FTMC, Baracat MM, Georgetti SR, Cardoso RD, Cunha TM, et al Flavonoids as Anti-Inflammatory and Analgesic Drugs: Mechanisms of Action and Perspectives in the Development of Pharmaceutical Forms In: Rahman AU, editor. Studies in Natural Products Chemistry. 1st ed Amsterdam: Elsevier; 2012 pp. 297–330.

[6] Pinho-Ribeiro FA, Zarpelon AC, Fattori V, Manchope MF, Mizokami SS, Casagrande R, et al Naringenin reduces inflammatory pain in mice. Neuropharmacology. 2016; 10.1016/j.neuropharm.2016.02.019 26907804

[7] Filho AW, Filho VC, Olinger L, de Souza MM. Quercetin: further investigation of its antinociceptive properties and mechanisms of action. Arch Pharm Res. 2008;31: 713–721. 10.1007/s12272-001-1217-2 18563352

[8] Valerio DA, Georgetti SR, Magro DA, Casagrande R, Cunha TM, Vicentini FT, et al Quercetin reduces inflammatory pain: inhibition of oxidative stress and cytokine production. J Nat Prod. 2009;72: 1975–1979. 10.1021/np900259y 19899776

[9] Huang J, Zhu M, Tao Y, Wang S, Chen J, Sun W, et al Therapeutic properties of quercetin on monosodium urate crystal-induced inflammation in rat. J Pharm Pharmacol. 2012;64: 1119–1127. 10.1111/j.2042-7158.2012.01504.x 22775215

[10] Azevedo MI, Pereira AF, Nogueira RB, Rolim FE, Brito GA, Wong DV, et al The antioxidant effects of the flavonoids rutin and quercetin inhibit oxaliplatin-induced chronic painful peripheral neuropathy. Mol Pain. 2013;9: 53. 24152430 10.1186/1744-8069-9-53 PMC3835704

[11] Maioli NA, Zarpelon AC, Mizokami SS, Calixto-Campos C, Guazelli CF, Hohmann MS, et al The superoxide anion donor, potassium superoxide, induces pain and inflammation in mice through production of reactive oxygen species and cyclooxygenase-2. Braz J Med Biol Res. 2015;48: 321–331. 10.1590/1414-431X20144187 25714890 PMC4418362

[12] Calixto-Campos C, Corrêa MP, Carvalho TT, Zarpelon AC, Hohmann MS, Rossaneis AC, et al Quercetin reduces Ehrlich tumor-induced cancer pain in mice. Anal Cell Pathol (Amst). 2015: 285708 10.1155/2015/285708 26351625 PMC4550761

[13] Anjaneyulu M, Chopra K. Quercetin, a bioflavonoid, attenuates thermal hyperalgesia in a mouse model of diabetic neuropathic pain. Prog Neuropsychopharmacol Biol Psychiatry. 2003;27: 1001–1005. 14499317 10.1016/S0278-5846(03)00160-X

[14] Nieman DC, Henson DA, Davis JM, Angela Murphy E, Jenkins DP, Gross SJ, et al Quercetin's influence on exercise-induced changes in plasma cytokines and muscle and leukocyte cytokine mRNA. J Appl Physiol (1985). 2007;103: 1728–1735. 17717114 10.1152/japplphysiol.00707.2007

[15] McAnulty SR, McAnulty LS, Nieman DC, Quindry JC, Hosick PA, Hudson MH, et al Chronic quercetin ingestion and exercise-induced oxidative damage and inflammation. Appl Physiol Nutr Metab. 2008;33: 254–262. 10.1139/H07-177 18347680

[16] Nieman DC, Henson DA, Maxwell KR, Williams AS, McAnulty SR, Jin F, et al Effects of quercetin and EGCG on mitochondrial biogenesis and immunity. Med Sci Sports Exerc. 2009;41: 1467–1475. 10.1249/MSS.0b013e318199491f 19516153

[17] O'Fallon KS, Kaushik D, Michniak-Kohn B, Dunne CP, Zambraski EJ, Clarkson PM. Effects of quercetin supplementation on markers of muscle damage and inflammation after eccentric exercise. Int J Sport Nutr Exerc Metab. 2012;22: 430–437. 22805422 10.1123/ijsnem.22.6.430

[18] Askari G, Ghiasvand R, Feizi A, Ghanadian SM, Karimian J. The effect of quercetin supplementation on selected markers of inflammation and oxidative stress. J Res Med Sci. 2012;17: 637–641. 23798923 PMC3685779

[19] Konrad M, Nieman DC, Henson DA, Kennerly KM, Jin F, Wallner-Liebmann SJ. The acute effect of ingesting a quercetin-based supplement on exercise-induced inflammation and immune changes in runners. Int J Sport Nutr Exerc Metab. 2011;21: 338–346. 21813917 10.1123/ijsnem.21.4.338

[20] Gao C, Chen X, Li J, Li Y, Tang Y, Liu L, et al Myocardial mitochondrial oxidative stress and dysfunction in intense exercise: regulatory effects of quercetin. Eur J Appl Physiol. 2014;114: 695–705. 10.1007/s00421-013-2802-9 24368555

[21] Borghi SM, Pinho-Ribeiro FA, Zarpelon AC, Cunha TM, Alves-Filho JC, Ferreira SH, et al Interleukin-10 limits intense acute swimming-induced muscle mechanical hyperalgesia in mice. Exp Physiol. 2015;100: 531–544. 10.1113/EP085026 25711612

[22] Cunha TM, Verri WA Jr, Vivancos GG, Moreira IF, Reis S, Parada CA, et al An electronic pressure-meter nociception paw test for mice. Braz J Med Biol Res. 2004;37: 401–407. 15060710 10.1590/s0100-879x2004000300018

[23] Ruiz-Miyazawa KW, Zarpelon AC, Pinho-Ribeiro FA, Pavão-de-Souza GF, Casagrande R, Verri Wa Jr. Vinpocetine reduces carrageenan-induced inflammatory hyperalgesia in mice by inhibiting oxidative stress, cytokine production and NF-κB activation in the paw and spinal cord. PLoS One. 2015;10: e0118942 10.1371/journal.pone.0118942 25822523 PMC4379066

[24] Hohmann MS, Cardoso RD, Pinho-Ribeiro FA, Crespigio J, Cunha TM, Alves-Filho JC, et al 5-lipoxygenase deficiency reduces acetaminophen-induced hepatotoxicity and lethality. Biomed Res Int. 2013: 627046 10.1155/2013/627046 24288682 PMC3832964

[25] Fattori V, Pinho-Ribeiro FA, Borghi SM, Alves-Filho JC, Cunha TM, Cunha FQ, et al Curcumin inhibits superoxide anion-induced pain-like behavior and leukocyte recruitment by increasing Nrf2 expression and reducing NF-kappaB activation. Inflamm Res. 2015;64: 993–1003. 10.1007/s00011-015-0885-y 26456836

[26] Zarpelon AC, Rodrigues FC, Lopes AH, Souza GR, Carvalho TT, Pinto LG, et al Spinal cord oligodendrocyte-derived alarmin IL-33 mediates neurophatic pain. FASEB J. 2016;30: 54–65. 10.1096/fj.14-267146 26310268

[27] Powers SK, Jackson MJ. Exercise-induced oxidative stress: cellular mechanism and impact on muscle force production. Physiol Rev. 2008;88: 1243–1276. 10.1152/physrev.00031.2007 18923182 PMC2909187

[28] Vicentini FT, He T, Shao Y, Fonseca MJ, Verri WA Jr, Fischer GJ, et al Quercetin inhibits UV irradiation-induced inflammatory cytokine production in primary human keratinocytes by suppressing NF-kappaB pathway. J Dermatol Sci. 2011;61: 162–168. 10.1016/j.jdermsci.2011.01.002 21282043

[29] Cleto LS, Oleto AF, Sousa LP, Barreto TO, Cruz JS, Penaforte CL, et al Plasma cytokine response, lipid peroxidation and NF-kB activation in skeletal muscle following maximum progressive swimminf. Braz J Med Biol Res. 2011;6: 546–552. 10.1590/s0100-879x2011007500050 21519639

[30] Seo MJ, Lee YJ, Hwang JH, Kim KJ, Lee BY. The inibitory effects of quercetin on obesity and obesity-induced inflammation by regulation of MAPK signalling. J Nutr Biochem. 2015;26: 1308–1316. 10.1016/j.jnutbio.2015.06.005 26277481

[31] Guazelli CF, Fattori V, Colombo BB, Georgetti SR, Vicentini FT, Casagrande R, et al Quercetin-loaded microcapsules ameliorate experimental colitis in mice by anti-inflammatory and antioxidant mechanism. J Nat Prod. 2013;76: 200–208. 10.1021/np300670w 23347547

[32] Carvalho TT, Borghi SM, Pinho-Ribeiro FA, Mizokami SS, Cunha TM, Ferreira SH, et al Granulocyte-colony stimulating factor (G-CSF)-induced mechanical hyperalgesia in mice: Role for peripheral TNFα, IL-1β and IL-10. Eur J Pharmacol. 2015;749: 62–72. 10.1016/j.ejphar.2014.12.023 25584775

[33] Faccioli LH, Souza GE, Cunha FQ, Poole S, Ferreira SH. Recombinant interleukin-1 and tumor necrosis factor induce neutrophil migration "in vivo" by indirect mechanisms. Agents Actions. 1990;30: 344–349. 2201176 10.1007/BF01966298

[34] Parameswaran N, Patial S. Tumor necrosis factor-alpha signaling in macrophages. Crit Rev Eukaryot Gene Expr. 2010;20: 87–103. 21133840 10.1615/critreveukargeneexpr.v20.i2.10 PMC3066460

[35] Gao Z, Zhang H, Liu J, Lau CW, Liu P, Chen ZY, et al Cyclooxygenase-2-dependent oxidative stress mediates palmitate-induced impairment of endothelium-dependent relaxations in mouse arteries. Biochem Pharmacol. 2014;91: 474–482. 10.1016/j.bcp.2014.08.009 25149102

[36] Verri WA Jr, Cunha TM, Parada CA, Poole S, Cunha FQ, Ferreira SH. Hypernociceptive role of cytokines and chemokines: targets for analgesic drug development? Pharmacol Ther. 2006;112: 116–138. 16730375 10.1016/j.pharmthera.2006.04.001

[37] Wang ZQ, Porreca F, Cuzzocrea S, Galen K, Lightfoot R, Masini E, et al A newly identified role for superoxide in inflammatory pain. J Pharmacol Exp Ther. 2004;309: 869–878. 14988418 10.1124/jpet.103.064154

[38] Ramyaa P, Krishnaswamy R, Padma VV. Quercetin modulates OTA-induced oxidative stress and redox signalling in HepG2 cells—up regulation of Nrf2 expression and down regulation of NF-kappaB and COX-2. Biochim Biophys Acta. 2014;1840: 681–692. 10.1016/j.bbagen.2013.10.024 24161694

[39] Schiavuzzo JG, Teixeira JM, Melo B, da Silva dos Santos DF, Jorge CO, Oliveira-Fusaro MC, et al Muscle hyperalgesia induced by peripheral P2X3 receptors is modulated by inflammatory mediators. Neuroscience. 2015;285: 24–33. 10.1016/j.neuroscience.2014.11.020 25446353

[40] Murase S, Terazawa E, Hirate K, Yamanaka H, Kanda H, Noguchi K, et al Upregulated glial cell line-derived neurotrophic factor through cyclooxygenase-2 activation in the muscle is required for mechanical hyperalgesia after exercise in rats. J Physiol. 2013;591: 3035–3048. 10.1113/jphysiol.2012.249235 23587883 PMC3832118

[41] Chen XL, Zhang Q, Zhao R, Medford RM. Superoxide, H2O2, and iron are required for TNF-alpha-induced MCP-1 gene expression in endothelial cells: role of Rac1 and NADPH oxidase. Am J Physiol Heart Circ Physiol. 2004;286: H1001–H1007. 14576080 10.1152/ajpheart.00716.2003

[42] Li Y, Reddy MA, Miao F, Shanmugam N, Yee JK, Hawkins D, et al Role of the histone H3 lysine 4 methyltransferase, SET7/9, in the regulation of NF-kappaB- dependent inflammatory genes. Relevance to diabetes and inflammation. J Biol Chem. 2008;39: 26771–26781. 10.1074/jbc.M802800200 PMC2546554 18650421

[43] Oeckinghaus A, Hayden MS, Ghosh S. Crosstalk in NF-κB signaling pathways. Nat Immunol. 2011;12: 695–708. 10.1038/ni.2065 21772278

[44] Ghosh S, Hayden MS. New regulators of NF-kappaB in inflammation. Nat Rev Immunol. 2008;8: 837–848. 10.1038/nri2423 18927578

[45] Hsu CC, Lien JC, Chang CW, Chang CH, Kuo SC, Huang TF. Yuwen02f1 suppresses LPS-induced endotoxemia and adjuvant-induced arthritis primarily through blockade of ROS formation, NFkB and MAPK activation. Biochem Pharmacol. 2013;85: 385–395. 10.1016/j.bcp.2012.11.002 23142712

[46] Bagul PK, Deepthi N, Sultana R, Banerjee SK. Resveratrol ameliorates cardiac oxidative stress in diabetes through deacetylation of NFkB-p65 and histone 3. J Nutr Biochem. 2015;26: 1298–1307. 10.1016/j.jnutbio.2015.06.006 26298192

[47] Nakao S, Ogata Y, Shimizu E, Yamazaki M, Furuyama S, Sugiya H. Tumor necrosis factor alpha (TNF-alpha)-induced prostaglandin E2release is mediated by the activation of cyclooxygenase-2 (COX-2) transcription via NFkappB in human gingival fibroblasts. Mol Cell Biochem. 2002;1–2: 11–18. 10.1023/a:1019927616000 12349897

[48] Marina R, Gonzalez P, Ferreras MC, Costilla S, Barrio JP. Hepatic Nrf2 expression is altered by quercetin supplementation in X-irradiated rats. Mol Med Rep. 2015;11: 539–546. 10.3892/mmr.2014.2741 25339115

[49] Akram M, Syed AS, Kim KA, Lee JS, Chang SY, Kim CY, et al Heme oxygenase 1-mediated novel anti-inflammatory activities of Salvia plebeia and its active components. J Ethnopharmacol. 2015;174: 322–330.51. 10.1016/j.jep.2015.08.028 26319962

[50] Jang M, Lee MJ, Choi JH, Kim EJ, Nah SY, Kim HJ, et al Ginsenoside Rb1 attenuates acute inflammatory nociception by inhibition of neuronal ERK phosphorilation by regulation of the Nrf2and NF-kB pathways. J Pain. 2016;17: 282–297. 10.1016/j.jpain.2015.10.007 26548970

[51] Xu XR, Yu HT, Yang Y, Hang L, Yang XW, Ding SH. Quercetin phospholipid complex significantly protects against oxidative injury in ARPE-19 cells associated with activation of Nrf2 pathway. Eur J Pharmacol. 2015;770: 1–8. 10.1016/j.ejphar.2015.11.050 26643168

[52] Hawke TJ, Garry DJ. Myogenic satellite cells: physiology to molecular biology. J Appl Physiol (1985). 2001;91: 534–551. 11457764 10.1152/jappl.2001.91.2.534

[53] Kang JS, Krauss RS. Muscle stem cells in developmental and regenerative myogenesis. Curr Opin Clin Nutr Metab Care. 2010;13: 243–248. 10.1097/MCO.0b013e328336ea98 20098319 PMC2872152

[54] Wang YX, Rudnicki MA. Satellite cells, the engines of muscle repair. Nat Rev Mol Cell Biol. 2012;13: 127–133. 10.1038/nrm3265 22186952

[55] Gao YJ, Ji RR. Chemokines, neuronal-glial interactions, and central processing of neuropathic pain. Pharmacol Ther. 2010;126: 56–68. 10.1016/j.pharmthera.2010.01.002 20117131 PMC2839017

[56] Souza GR, Talbot J, Lotufo CM, Cunha FQ, Cunha TM, Ferreira SH. Fractalkine mediates inflammatory pain through activation of satellite glial cells. Proc Natl Acad Sci U S A. 2013;110: 11193–11198. 10.1073/pnas.1307445110 23776243 PMC3704031

[57] Grau JW, Huie JR, Lee KH, Hoy KC, Huang YJ, Turtle JD, et al Metaplasticity and behavior: how training and inflammation affect plastic potential within the spinal cord and recovery after injury. Front Neural Circuits. 2014;8: 100 10.3389/fncir.2014.00100 25249941 PMC4157609

[58] Liu XD, Wang JJ, Sun L, Chen LW, Rao ZR, Duan L, et al Involvement of medullary dorsal horn glial cell activation in mediation of masseter mechanical allodynia induced by experimental tooth movement. Arch Oral Biol. 2009;54: 1143–1150. 10.1016/j.archoralbio.2009.09.006 19853838

[59] Chacur M, Lambertz D, Hoheisel U, Mense S. Role of spinal microglia in myositis-induced central sensitisation: an immunohistochemical and behavioural study in rats. Eur J Pain. 2009;13: 915–923. 10.1016/j.ejpain.2008.11.008 19095475

[60] Tenschert S, Reinert A, Hoheisel U, Mense S. Effects of a chronic myositis on structural and functional features of spinal astrocytes in the rat. Neurosci Lett. 2004;361: 196–199. 15135927 10.1016/j.neulet.2003.12.003

[61] Zhao YJ, Liu Y, Li Q, Zhao YH, Wang J, Zhang M, et al Involvement of trigeminal astrocyte activation in masseter hyperalgesia under stress. Physiol Behav. 2015;142: 57–65. 10.1016/j.physbeh.2015.02.005 25660342

[62] Ding Y, Chang C, Xie L, Chen Z, Ai H. Intense exercise can cause excessive apoptosis and synapse plasticity damage in rat hippocampus through Ca(2)(+) overload and endoplasmic reticulum stress-induced apoptosis pathway. Chin Med J (Engl). 2014;127: 3265–3271. 25266525

[63] Kiyomoto M, Shinoda M, Okada-Ogawa A, Noma N, Shibuta K, Tsuboi Y, et al Fractalkine signaling in microglia contributes to ectopic orofacial pain following trapezius muscle inflammation. J Neurosci. 2013;33: 7667–7680. 10.1523/JNEUROSCI.4968-12.2013 23637160 PMC6618944

[64] Wang D, Couture R, Hong Y. Activated microglia in the spinal cord underlies diabetic neuropathic pain. Eur J Pharmacol. 2014;728: 59–66. 10.1016/j.ejphar.2014.01.057 24508519

[65] Wiczkowski W, Skipor J, Misztal T, Szawara-Nowak D, Topolska J, Piskula MK. Quercetin and isorhamnetin aglycones are the main metabolites of dietary quercetin in cerebrospinal fluid. Mol Nutr Food Res. 2015;59: 1088–1094. 10.1002/mnfr.201400567 25727325

[66] Testa G, Gamba P, Badilli U, Gargiulo S, Maina M, Guina T, et al Loading into nanoparticles improves quercetin's efficacy in preventing neuroinflammation induced by oxysterols. PLoS One. 2014;9: e96795 10.1371/journal.pone.0096795 24802026 PMC4011877

[67] Faria A, Meireles M, Fernandes I, Santos-Buelga C, Gonzalez-Manzano S, Dueñas M, et al Flavonoid metabolites transport across a human BBB model. Food Chem. 2014;149: 190–196. 10.1016/j.foodchem.2013.10.095 24295694

[68] Yang T, Kong B, Gu JW, Kuang YQ, Cheng L, Yang WT, et al Anti-apoptotic and anti-oxidative roles of quercetin after traumatic brain injury. Cell Mol Neurobiol. 2014;34: 797–804. 10.1007/s10571-014-0070-9 24846663 PMC11488927

[69] Diaz-Ruiz A, Alcaraz-Zubeldia M, Maldonado V, Salgado-Ceballos H, Mendez-Armenta M, Rios C. Differential time-course of the increase of antioxidant thiol-defenses in the acute phase after spinal cord injury in rats. Neurosci Lett. 2009;452: 56–59. 10.1016/j.neulet.2009.01.020 19159657

[70] Guedes RP, Dal Bosco L, Araújo AS, Belló-Klein A, Ribeiro MF, Partata WA. Sciatic nerve transection increases gluthatione antioxidant system activity and neuronal nitric oxide synthase expression in the spinal cord. Brain Res Bull. 2009;80: 422–427. 10.1016/j.brainresbull.2009.08.007 19683561

